# Dissociating Cerebellar Regions Involved in Formulating and Articulating Words and Sentences

**DOI:** 10.1162/nol_a_00148

**Published:** 2024-08-15

**Authors:** Oiwi Parker Jones, Sharon Geva, Susan Prejawa, Thomas M. H. Hope, Marion Oberhuber, Mohamed L. Seghier, David W. Green, Cathy J. Price

**Affiliations:** Wellcome Centre for Human Neuroimaging, University College London, London, UK; Department of Engineering Science, University of Oxford, Oxford, UK; Jesus College, University of Oxford, Oxford, UK; Centre for Mind and Behaviour, Anglia Ruskin University, Cambridge, UK; Healthcare Engineering Innovation Center (HEIC), Biomedical Engineering Department, Khalifa University of Science and Technology, Abu Dhabi, UAE; Experimental Psychology, University College London, London, UK

**Keywords:** cerebellum, cortex, fMRI, picture description, sentence processing, speech production

## Abstract

We investigated which parts of the cerebellum are involved in formulating and articulating sentences using (i) a sentence production task that involved describing simple events in pictures; (ii) an auditory sentence repetition task involving the same sentence articulation but not sentence formulation; and (iii) an auditory sentence-to-picture matching task that involved the same pictorial events and no overt articulation. Activation for each of these tasks was compared to the equivalent word processing tasks: noun production, verb production, auditory noun repetition, and auditory noun-to-picture matching. We associate activation in bilateral cerebellum lobule VIIb with sequencing words into sentences because it increased for sentence production compared to all other conditions and was also activated by word production compared to word matching. We associate a paravermal part of right cerebellar lobule VIIIb with overt motor execution of speech, because activation was higher during (i) production and repetition of sentences compared to the corresponding noun conditions and (ii) noun and verb production compared to all matching tasks, with no activation relative to fixation during any silent (nonspeaking) matching task. We associate activation within right cerebellar Crus II with covert articulatory activity because it activated for (i) all speech production more than matching tasks and (ii) sentences compared to nouns during silent (nonspeaking) matching as well as sentence production and sentence repetition. Our study serendipitously segregated, for the first time, three distinct functional roles for the cerebellum in generic speech production, and it demonstrated how sentence production enhanced the demands on these cerebellar regions.

## INTRODUCTION

The goal of this study was to better understand the role of the cerebellum during simple sentence production. In everyday life we routinely use simple sentences to describe a scene or ongoing event (e.g., “the cat is licking the spoon”). Cerebellar involvement in such processing is well recognised (Dien et al., 2003; Jeong et al., 2007; Strelnikov et al., 2006; Uchiyama et al., 2008). For example, sentence production impairments have been associated with cerebellar pathology (Fabbro et al., 2004; Gasparini et al., 1999; Guell et al., 2015; Justus, 2004; Karaci et al., 2008; Leggio et al., 2008; Murdoch & Whelan, 2007; Silveri et al., 1994; Zettin et al., 1997). As these deficits are often subtle and cannot be observed using routine diagnostic tools (Mariën et al., 2014), there is currently insufficient data to understand the precise location of the cerebellar lesion sites associated with sentence production impairments. There is also a paucity of evidence from functional imaging studies because the bulk of research on sentence processing has not concerned sentence production (see meta-analyses by Keren-Happuch et al., 2014; Stoodley & Schmahmann, 2009). Instead, it has focused on the involvement of the cerebellum during sentence comprehension (King et al., 2019; Lesage et al., 2017; Miall et al., 2016; Moberget et al., 2014; Skipper & Lametti, 2021).

### The Present Study

**Figure 1.**
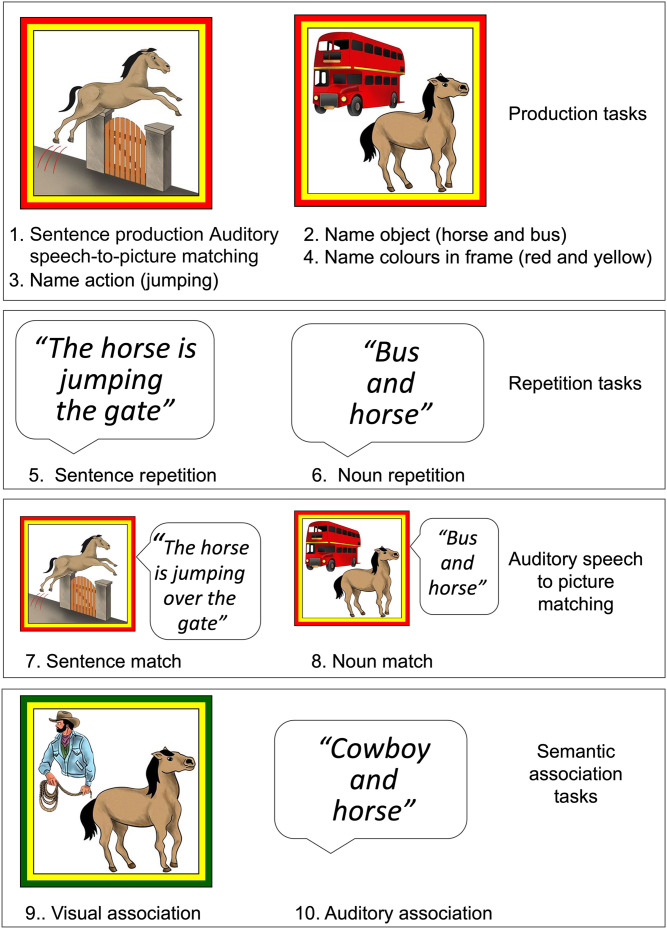
Examples of stimuli used for the 10 different tasks. The paradigm consists of 10 tasks including production tasks (1–4), auditory repetition tasks (5 and 6), auditory to picture matching tasks (7 and 8), and semantic association tasks (9 and 10). The names of the tasks, referred to in the text are (1) sentence production, (2) noun production, (3) verb production (name action), (4) colour production, (5) sentence repetition, (6) noun repetition, (7) auditory sentence-to-picture matching, (8) auditory noun to picture matching, (9) visual semantic association, and (10) auditory semantic association. The two colours in the frames around all pictures are the colours that need to be named in the colour naming task only.

We used functional magnetic resonance imaging (fMRI) of healthy participants to investigate how the cerebellum responded when participants were shown pictures of events that included two objects that were interacting in one of four possible ways (jumping, falling, eating, or drinking; see Figure 1). Participants were instructed to describe the event in a simple declarative sentence (“The horse is jumping over the gate”; “The goat is eating the hat”; “The cat is drinking from the jug”; “The camera is falling from the chair”). The names, verbs, and sentence structure were therefore tightly constrained. Even this very simple sentence production task involves multiple types of processing. The focus of the current article is on (i) sentence formulation and (ii) sentence articulation. We define *sentence formulation* as the processes involved in (i) finding a syntactic structure that assigns the roles of the two objects in the event to the roles of grammatical subject and grammatical object and (ii) ordering the words to capture the intended meaning (e.g., “The goat is eating the hat,” but not “The hat is eating the goat”). *Sentence articulation*, in contrast, refers to the processes involved in the planning and motor control of speech output at the sentence level.

To dissociate sentence formulation from sentence articulation, we included two other sentence processing tasks: auditory sentence repetition and auditory sentence-to-picture matching. *Auditory sentence repetition* entailed articulatory planning and motor execution of sentences with low demands on sentence formulation because the word order is specified by the heard sentence. *Auditory sentence-to-picture matching* entailed conceptualising the event in a picture (as in sentence production) and indicating whether this event matches that in a simultaneously heard sentence, using a finger press response. This sentence matching task explicitly involves recognising the relationship between objects and verbs (event conceptualisation) and may also implicitly involve a low level of sentence formulation and articulatory planning, even though there is no motor execution of speech. We refer to this as *implicit* sentence formulation and articulation because we are implying that it is occurring versus directly observing that it has occurred. In contrast, we refer to the *explicit* involvement of sentence formulation and articulation in sentence production; and likewise, the explicit involvement of articulatory planning in sentence repetition.

### Predictions

We predicted that sentence formulation would activate the cerebellum because cerebellar lesions have been associated with impairments in grammar (agrammatism) or sequencing (Leggio et al., 2008) in both case studies (Fabbro et al., 2000, 2004; Gasparini et al., 1999; Murdoch & Whelan, 2007; Silveri et al., 1994; Zettin et al., 1997) and group studies (Justus, 2004; Karaci et al., 2008). Patients with unilateral cerebellar lesions have also been shown to lack the P600 component which reflects sensitivity to syntactic violations, although their performance on a simple syntactic violation detection task was normal (Adamaszek et al., 2012). We are not able to predict which parts of the cerebellum are involved in sentence formulation because (i) lesion site varied within and across the aforementioned studies and (ii) there is a lack of data from functional imaging studies that dissociated sentence formulation from speech articulation.

We predicted that articulating sentences compared to nouns or verbs would enhance activation in the primary motor representation in bilateral cerebellar lobule VI (Guell et al., 2018), which has been most consistently associated with articulation in functional imaging studies of neurotypical adults (see, e.g., Bohland & Guenther, 2006; Correia et al., 2020; Geva et al., 2021; Grabski et al., 2012; Peeva et al., 2010). Although less consistently, articulation has also been associated with the secondary motor representation (Guell et al., 2018) in bilateral lobule VIIIa (Bohland & Guenther, 2006; Brown et al., 2009; Errante & Fogassi, 2020; Geva et al., 2021; Nota & Honda, 2004) and lobule VII/Crus I (Bohland & Guenther, 2006; Geva et al., 2021; Ghosh et al., 2008; Shuster & Lemieux, 2005). These regions are more likely to be activated when task demands are high (Guell et al., 2018; King et al., 2019) as expected for the articulation of sentences compared to nouns or verbs.

### Interpreting Sentence Level Activation

**Figure 2.**
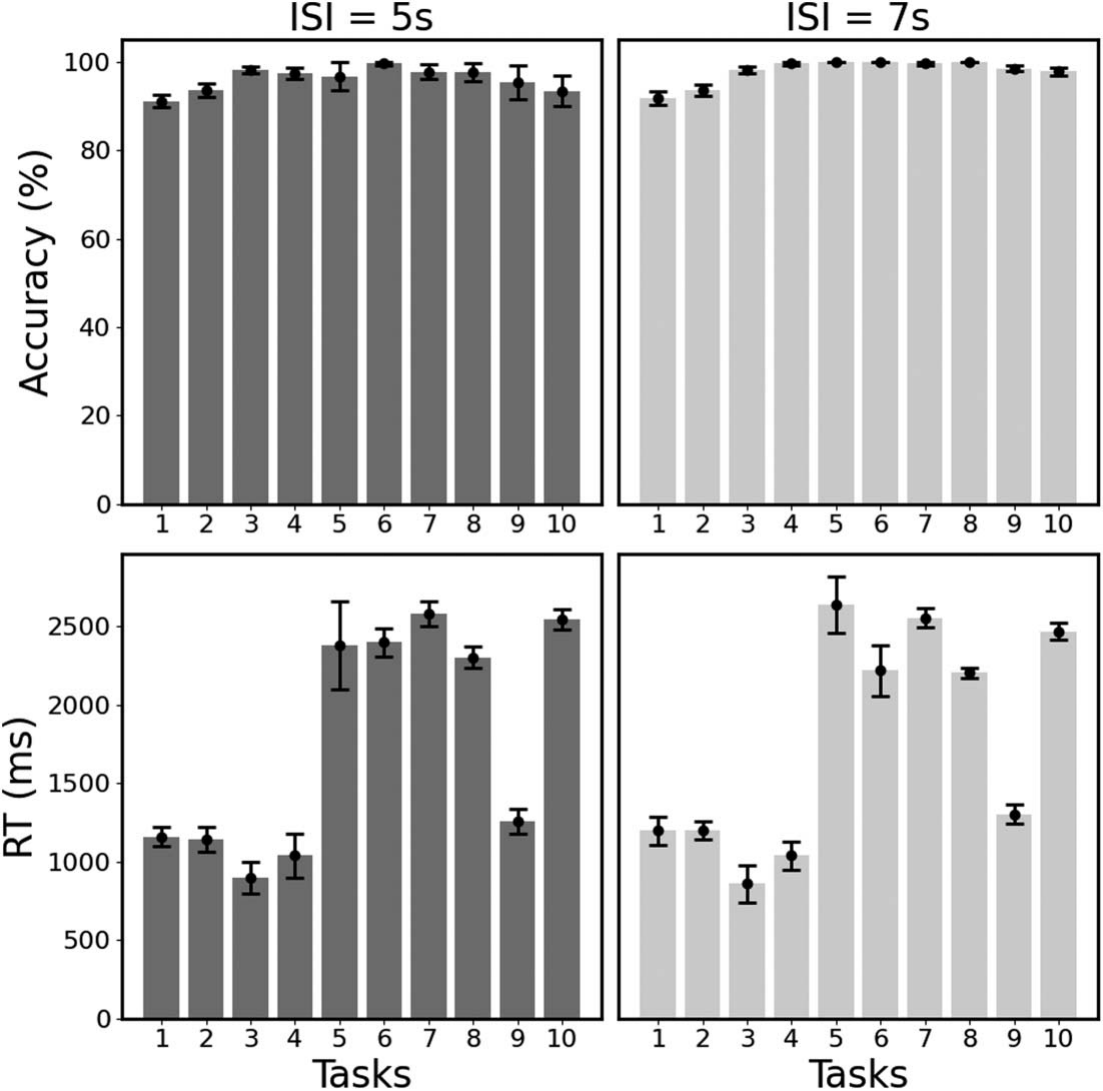
Behavioural results. Accuracy (in percent, top panel) and response time (RT, in ms, bottom panel) are shown for data collected with 5 s and 7 s interstimulus intervals (ISIs). In both cases, mean accuracy was over 90% for all tasks. Note that speaking RTs were only extracted from 82% of the speaking conditions and are therefore interpreted with caution. See text for further details and Figure 1 for task numbers.

**Figure 3.**
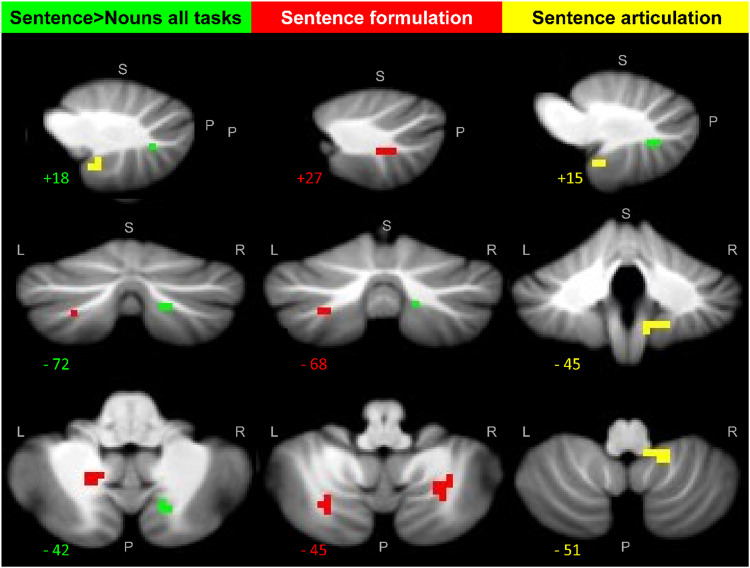
Cerebellar regions that were more activated by sentences than nouns. This figure displays, on the SUIT template (Diedrichsen, 2006), the cerebellar regions where activation was enhanced by sentences. Green shows the right cerebellar Crus II region that was more activated for sentences than nouns across task. Red shows the bilateral cerebellar lobule VII regions that were more activated for sentence than noun production but not for sentence more than nouns during the repetition and matching tasks. Yellow shows the cerebellar lobule VIIIb region that was more activated for sentences than nouns in the speaking conditions (production and repetition) but not the matching conditions. The top row shows sagittal slices, the middle row shows coronal slices, and the bottom row shows axial slices. The numbers correspond to the MNI co-ordinates of each slice. L = left, R = right, S = superior, P = posterior.

**Figure 4.**
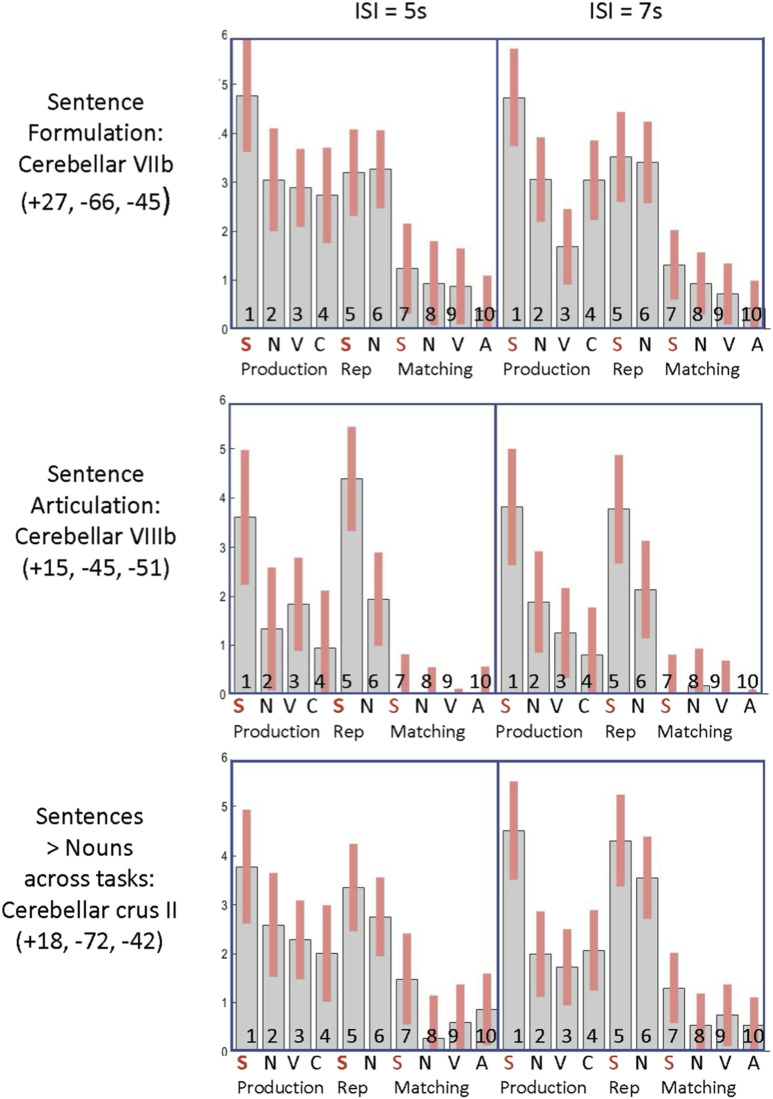
Effect sizes, across conditions, for cerebellar regions associated with sentence processing. This figure illustrates the effect sizes for each condition in the cerebellar regions that were enhanced by sentences (effect sizes refer to the estimated parameters of a general linear model). The left side of the plot shows effects from the participants in the version with an ISI of 5 s. The right side shows effects from the participants in the version with an ISI or 7 s. This is to illustrate that the effects were replicated across ISI and were not driven by one. Top row shows the responses in the right cerebellar lobule VII region that was more activated for sentence than nouns during production but not repetition and matching (i.e., the response was consistent with sentence formulation). Middle row shows the responses in the right cerebellar lobule VIIIb region that was more activated for sentences than nouns in the speaking conditions (production and repetition) but not the matching conditions. Bottom row shows the responses in the right cerebellar Crus II region that was more activated for sentences than nouns across task. Numbers on each effect correspond to the 10 tasks illustrated in Figure 1. S = sentences, N = nouns, V = verbs, C = colour condition, V = visual semantic association matching, A = auditory semantic association matching. The first four tasks are production tasks (with picture stimuli), Tasks 5–6 are auditory repetition (Rep) and the last four tasks are matching tasks (no speech).

**Figure 5.**
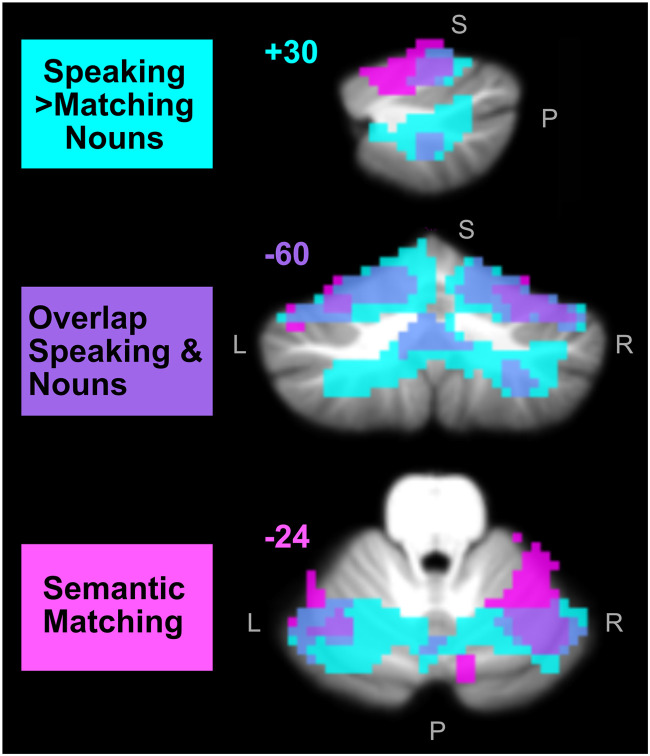
Cerebellar activation identified in the speaking and semantic localisers. This figure displays, on the SUIT template (Diedrichsen, 2006), the cerebellar regions that were enhanced by speaking (localiser 6 = cyan) and semantics (localiser 3 = pink), with the overlap showing in purple. The sentence processing effects shown in Figure 3 were all within the speaking regions but did not overlap with the semantic regions. The top row shows a sagittal slice, the middle row shows a coronal slice, and the bottom row shows axial slices. The numbers correspond to the MNI coordinates of each slice. L = left, R = right, S = rsuperior, P = posterior.

To control for other processes involved in our three sentence processing tasks, our experimental design included seven other tasks that did not involve sentence formulation or articulation (see Figure 1 and Table 1). We focused on activation that was higher for sentence production than noun or verb production. This might be indicative of a sentence specific function. Alternatively, it might signify the additional demands that sentence processing places on other lower level functions that are not specific to sentence formulation or articulation. To weigh up evidence for these different interpretations, we functionally localise activation associated with six different types of processing: (1) object recognition and noun retrieval during noun production (task 2 in Figure 1); (2) event conceptualisation and verb retrieval during verb production (task 3 in Figure 1); (3) semantic association (tasks 9 and 10 in Figure 1), (4) auditory short-term memory during auditory semantic associations (task 9 in Figure 1), (5) visual short-term memory during visual semantic associations (task 10 in Figure 1), and (6) overt speaking including articulatory planning and the motor execution of speech (tasks 2, 3, 4, and 6 in Figure 1). We then looked to see whether activation associated with any of these processes overlapped with activation associated with sentence formulation or articulation. Table 1 shows which tasks were used to identify each function of interest; see statistical contrasts in Materials and Methods for more details.

**Table 1.** Tasks used to identify processes of interest and functional localisers

Task number	1	2	3	4	5	6	7	8	9	10
Task type	Production				Repetition		AV Matching		Semantic	
Stimulus type	S	N	Vb	C	S	N	S	N	V	A
Sentence processing of interest										
Sentence formulation	E						I			
Sentence articulation	E				E		I			
Functional localisers (nonsentence tasks only)										
1. Object recognition & noun retrieval		E								
2. Event conceptualisation			E							
3. Semantic association									E	E
4. Auditory short-term memory										E
5. Visual short-term memory									E	
6. Speaking (articulating) nouns		E	E	E		E				

*Note*. See Figure 1 to link the 10 task numbers to the 10 task names. S = sentences, N = nouns, Vb = verbs, C = colour, V = visual (picture) stimuli, A = auditory (speech) stimuli. E = explicitly involved in the task; I = may occur implicitly.

## MATERIALS AND METHODS

The study was approved by the London Queen Square Research Ethics Committee. All participants gave written informed consent prior to scanning and received financial compensation for their time.

### Participants

The data from this experiment were acquired from 25 right-handed participants (15 female, 10 male; aged 23–37 years with a mean and standard deviation of 30.35 ± 3.90 years). Handedness was determined using the Edinburgh Handedness Inventory (Oldfield, 1971).

### Task Details

There were 10 tasks in total, which are illustrated in Figure 1. The tasks involved visual, audio, or both visual and audio stimuli, and required spoken or manual responses (button presses) from the participants. Additional task details can be found in Gajardo-Vidal et al. (2018).

Task 1 (sentence production) required participants to describe a picture. The pictures were coloured drawings of situations with two interacting objects (e.g., a goat, a hat, and an asymmetric action relating them such as eating). Participants were instructed to speak aloud using active sentences of the form *the noun is verbing the noun* (e.g., “The goat is eating the hat”) or *the noun is verbing preposition the noun* (“The zebra is drinking from the pool”) and to limit themselves to using only one of four verbs in each sentence (i.e., eat, drink, jump, or fall). We piloted the instructions and stimuli using a separate group of fluent English-speaking participants (*n* = 11, who did not participate in the fMRI experiment) to ensure that the task could be completed with low variance in the responses. This cohort was also involved in piloting of additional aspects of the study (see Task 9).

Task 2 (noun production) was similar to sentence production, except that the pictures depicted two objects, not interacting, and the participants were instructed to name both objects aloud (e.g., clock and pumpkin). The use of the conjunction “and” meant that participants produced noun phrases in this task, while also increasing the length of the spoken responses to better match to the responses in sentence production.

Task 3 (verb production) used the same kind of stimuli as sentence production (pictures of events). However, in this task participants were instructed just to name aloud the action by producing the relevant verb in gerund form (e.g., eating). Correct response in this task entails that participants have understood the event (conceptualised it) and provided explicit evidence of this conceptualisation.

In Task 4 (colour production), participants named aloud the two colours bordering the picture (e.g., orange and green). The stimuli were of the same type as those in the noun production task. However, while the pictures in all the tasks with visual stimuli included a coloured border, participants were only required to attend to it in this task. The format of response (*colour and colour*) was also grammatically similar to the response pattern required by noun production (*noun and noun*). We expected this task to identify brain areas involved in word retrieval without requiring explicit recognition or naming of the objects in the picture.

In Task 5 (sentence repetition) participants heard a spoken sentence (e.g., “The goat is eating the hat”), having been instructed to repeat it aloud. Participants were further asked to keep their eyes open and attend to a fixation cross during this task.

In Task 6 (noun repetition), participants heard noun phrases like “clock and pumpkin” and were required to repeat the phrases aloud. The task otherwise resembled the sentence repetition task.

Task 7 (auditory sentence-to-picture matching) required participants to determine whether a heard sentence matched the event depicted in a concurrently presented picture. For example, if they saw a picture of the goat eating the hat and heard “The goat is eating the hat,” they were to press the “match” button; otherwise, they were to press the “not match” button. In non-matching trials (1 in 4), the only change was to the verb used in the heard sentence. For example, hearing “The goat is falling on the hat” while seeing a picture of the goat eating the hat. Participants indicated their response by pressing one of two buttons with the index or middle finger on their right hand.

Task 8 (auditory noun-to-picture matching) involved paired audio and visual stimuli (as in the auditory sentence-to-picture matching task) but the stimuli were two nouns in a phrase (e.g., “shelf and carrot”) instead of sentences. In the non-matching trials (1 in 4), one of the object names was changed in the heard names (e.g., “shelf and nest”) with the incorrect name occurring first or second an equal number of times.

Task 9 (visual semantic association) required participants to make a semantic decision about two depicted objects. Half of the pairs were semantically related (e.g., “door and key”) while the other half were unrelated (“clock and pumpkin”). Participants indicated their response by pressing one of two buttons with the index or middle fingers on their right hand. Semantic relatedness was determined in a pilot experiment in which we asked the piloting cohort (see Task 1) to judge the relatedness of entity pairs. Only pairs that were consistently judged to be related, or consistently judged to be unrelated, were used in the main experiment.

Finally, Task 10 (auditory semantic association) was the same as the visual association task but used auditory rather than visual stimuli. As with the other auditory tasks, participants were asked to keep their eyes open and focus on a fixation cross throughout the task.

#### Counterbalancing tasks within participants

Within participant, the order of six speaking (Sp) and four Finger press (F) task responses was either [Sp-Sp-Sp-Sp-Sp-Sp–F-F-F-F] or [F-F-F-F–Sp-Sp-Sp-Sp-Sp-Sp]. We did not attempt to balance response mode further because flipping between speaking and finger pressing was expected to enhance head movement. The order of production (P) and repetition (R) tasks was either P-R-P-R or R-P-R-P; the order of verb production (V) and colour (C) production was either VC or CV; the order of sentences (S) and nouns (N) was either S-N-N-S or N-S-S-N; the order of auditory-picture matching (APM) and semantic associations (SA) was APM-SA-SA-APM; the order of visual (V) or auditory (A) semantic association matching was either V-A or A-V.

Within these counterbalancing constraints, two possible task orders were generated. Task order 1 was: (i) auditory sentence repetition, (ii) noun production, (iii) noun repetition, (iv) sentence production, (v) verb production, (vi) colour production, (vii) auditory sentence-to-picture matching, (viii) visual semantic association, (ix) auditory semantic association, and (x) auditory noun-to-picture matching. Task order 2 was the exact reversal of the first task order (x to i).

#### Stimulus selection and creation

First, the sentence stimuli were created by selecting familiar animals (e.g., monkey, zebra, deer), humans in various roles (e.g., king, nurse, clown), and objects (e.g., glove, book, potato) that could be recognised easily when presented visually, using high-definition coloured line drawings drawn by a professional artist; named easily because the item was highly familiar; and pictured in an event that involved one of four verbs (“eating,” “drinking,” “falling,” and “jumping”) with another familiar object that was sufficiently large to be seen in the picture. Examples include a monkey eating a banana, a king drinking from a cup, a glove falling from a bag, and a deer jumping over a fence. In total, we created 60 different sentences (15 for each of the 4 verbs) using 120 pictures of different objects meeting the constraints on the verbs, with no repetition of any object across sentences. All 11 participants in the pilot cohort agreed on the verb implicit in the picture in all cases. The position of the object acting as the agent in each event varied in whether its position was on the left or right of the object acted upon.

Second, semantic associations were created by rearranging the 120 objects into 30 semantically related pairs (e.g., “pirate and boat”) and 30 semantically unrelated pairs (e.g., “frog and plate”). The pairing in this case was constrained because all new pairs (semantically related or unrelated) were not the same as the pairs of objects within any of the sentences. The 60 sentences (each involving a pair of objects) was split into six sets of 10 sentences (20 objects in total). Within each set, the 20 objects were rearranged into five semantically related pairs and five semantically unrelated pairs. The pilot testing established full agreement on whether each pair was semantically related or not.

Third, each set of 20 objects was rearranged a third time into 10 different unrelated pairs, none of which corresponded to the sentence pairs or the semantically related or unrelated pairs. Across each of the three groupings, each object was therefore presented in three different pairs. For example, banana was presented as “monkey eating a banana” (sentence), “apple and banana” (semantically related) and “snake and banana” (semantically unrelated). This resulted in 180 different object pairs (3 pairings × 10 pairs × 6 sets). Coloured drawings of each object pair (see Figure 1) were created by a professional artist (Eldad Druks). Auditory speech associated with each pair (see Figure 1) was recorded by a speaker of Standard Southern British English.

The 60 sentence pairs were presented in sentence production, verb production, sentence repetition, and auditory sentence-to-picture matching (Tasks 1, 3, 5, and 7 in Figure 1). The unrelated pairs were presented in noun production, colour naming, auditory noun repetition, and auditory word-to-picture matching (Tasks 2, 4, 6 and 8 in Figure 1). The semantic association pairs were presented in Tasks 9 and 10 in Figure 1.

#### Counterbalancing stimuli across tasks

For each of the 10 tasks, we presented 10 different pairs of stimuli (100 pairs in total). Ensuring that no participant was presented with the same object twice would have required 200 different objects. As we only identified 120 objects that met all our criteria for selection (see above), each participant was presented with 80 objects twice and the other 40 objects once. Repetition of an object was very carefully controlled within and across participants. Compared to the first presentation, the second presentation was always in a different (i) pair and (ii) task and (iii) stimulus modality, that is, if an object was first presented as a picture, it was re-presented in the auditory domain. No object name was articulated (spoken by the participant) more than once. If an object was named in one articulation condition (production or repetition), we re-presented it in a matching task, that does not require speech production. No object was seen in both the visual and auditory semantic association conditions (to avoid conflict in semantic associations).

#### Counterbalancing stimuli and tasks across participants

Each object repeated twice across three participants. For example, one participant has a repeat of sets 1, 2, 3, 4; a second participant has a repeat of sets 5, 6, 1, 2; and a third participant has a repeat of sets 3, 4, 5, 6. However, to additionally control for task orders (see above) as well as stimulus repetition, six participants were needed (3 to repeat the objects twice to counterbalance the task orders).

### In-Scanner Procedure

Visual stimuli were presented via an LCD projector and an adjustable head-coil mirror onto a screen that was clearly visible to the participant. Each stimulus was scaled to 350 pixels^2^, subtending a visual angle of 7.4° with a screen resolution of 1024 × 768. Auditory stimuli were presented via MRI compatible headphones (MR Confon, Magdeburg, Germany) which filtered out ambient in-scanner noise. The duration of each auditory stimulus ranged from 1.76 s to 2.5 s. (Sentence stimuli had longer durations than word stimuli because they included more words.) Volume levels were adjusted to suit each participant before data acquisition. During auditory trials, participants kept their eyes open and fixated on a central cross. For the production tasks, spoken responses were recorded via a noise-cancelling MRI microphone (FORMI II Optoacoustics, Or-Yehuda, Israel). These auditory recordings were then transcribed manually for offline analysis to record in-scanner accuracy. For matching tasks, participants used two fingers of their right hand to press one of two buttons on an fMRI compatible button box to indicate “match” or “not match.” The assignment of buttons to responses was counterbalanced across participants.

Prior to scanning, participants were trained on an independent set of stimuli until they could perform the tasks without error. Once in the scanner, participants performed each of the 10 tasks, one after another in separate sessions (runs). Each session started with the visual display “Get Ready” for 15.4 s, while five dummy scans were acquired as the scanner equilibrated. This was followed by five blocks of stimuli alternating with blocks of “fixation,” which involved resting with eyes open with attention on a fixation cross. Each block included four stimuli with two objects per stimulus (40 objects presented per task). Each block of stimuli was preceded by 3.08 s of visually presented instructions that served to inform (block 1) or remind (blocks 2–5) the participants of the current task.

Stimuli were presented at one of two different interstimulus intervals (ISIs): Either 5 s or 7 s. Using two different ISIs allowed us to replicate the experiment over two different groups and showed that the paradigm can be used in different circumstances. For example, one might want a longer ISI for studies of stroke patients who have difficulty with sentence production and a shorter ISI when time in the scanner is the most important issue. For the 12 participants in the ISI = 5 s group, six participants performed the task in the first order (starting with speech) and six performed the task in the second order (starting with matching), with stimuli fully rotated across conditions for each group (as explained above). For the 13 participants in the ISI = 7 s group, six performed the task in first order and seven performed the task in the second order, with stimuli fully rotated across conditions for each group of six. As illustrated in the Results section, none of the effects of interest was influenced by the ISI length. Details of all other presentation details are provided in Table 2.

**Table 2.** Summary of stimulus presentation and scanning parameters

Participant group	ISI = 5 s	ISI = 7 s
Number of participants	12	13
Number of runs/sessions	10	10
Tasks per run	1	1
Blocks per run	5	5
Object pairs per block	4	4
Objects per block	8	8
Block instructions (s)	3.08	3.08
Block duration (s)	20	28
Visual stimulus duration (s)	2.5	2.5
Auditory stimulus duration (s)	1.76–2.5	1.76–2.5
Interstimulus time (s)	5	7
Fixation block duration (s)	16.96	18.20
Run duration (minutes)	3.4	4.1
Scanning parameters		
Repetition time	3.08	3.08
Number of pre-block dummies	5	5
Dummy scan time (s)	15.4	15.4
Number of slices per image	44	44
Total number of images	61	85

*Note*. ISI = interstimulus interval.

### Analysis of In-Scanner Behaviour

For the matching tasks, the accuracy and speed of response was measured by button presses. A response was categorised as “correct” if it matched the expected target and as “incorrect” if the response was missing or did not match the target.

For the speech production tasks, the accuracy and response times (RTs) were measured from audio recordings of the spoken response. These audio recordings were transcribed, checked for errors, and subjected to a signal processing analysis that automatically extracted the spoken RTs for each stimulus (see below). For the sentence production and noun production tasks a trial was considered to be correct if >10% of other participants made the equivalent response, even if it was not the same as our intended target (e.g., if >10% of participants said “mug” when our expected response was “cup”). Only correct trials were used in the fMRI analyses.

RTs for correct spoken responses were measured using an adaptive-window-moving-average filter that was customised to remove noise for each participant. The optimal window length (i.e., the width of the maximally smoothed audio stream) was based on a sample of the audio file collected at baseline before any stimuli were presented. Once the whole audio recording was smoothed to remove high-frequency noise, we defined the onset of speech as the first rise in absolute amplitude above one standard deviation from the mean amplitude of a stimulus event. This process was only successful for 123/150 = 82% of all speaking conditions (150 from 25 subjects × 6 speaking conditions). Speaking RT data were fully or partially missing from 10/25 participants (5 participants when ISI = 5 s and 5 participants when ISI = 7 s). The relationship between speaking RTs and brain activation is therefore considered with caution.

Differences in spoken RTs were analysed in a 2 × 3 repeated-measures analysis of variance (ANOVA) with ISI (5 s vs. 7 s ISI) as a between-participants factor and syntax-related task (sentence naming or object naming vs. verb naming) as a within-participants factor. Interpreting RTs in the repetition tasks was complicated by the variable duration of auditory stimuli and was not of primary interest (though see Figure 2 in the Results for an overview of all RT results). We used Mauchly’s test of sphericity to evaluate the equality of variance between spoken RT samples, employing standard corrections when the assumption of sphericity was violated. That is, we applied unequal variance *t* tests and Huynh–Feldt corrected *F* ratios (Mauchly’s Σ between 1 and 0.75). For one participant (in the 5 s ISI group), behavioural data were missing for both semantic association tasks.

### MRI Data Acquisition

Structural and functional data were acquired on a 3T scanner (Trio, Siemens Medical Systems, Erlangen, Germany) using a 12-channel head coil. Head movement was constrained by adding padding around the head mould. We also corrected for movement artefacts during data preprocessing, as described below and examined the degree of head movement measured for each participant. Functional images consisted of a gradient-echo EPI (echo planar imaging) sequence and 3 × 3 mm in-plane resolution (repetition time [TR]/echo time [TE]/flip angle = 3,080 ms/30 ms/90°, field of view = 192 mm, matrix size = 64 × 64, 44 slices, slice thickness = 2.5 mm, and interslice gap = 0.5 mm). Although statistical power may have been increased if data acquisition was limited to the cerebellum, our goal here is to assess the cerebellar responsivity within a whole brain paradigm—and, as reported in the results, the protocol was sufficient to identify several novel and highly significant effects.

For the 5 s ISI group (12 participants), we acquired 61 image volumes per task (3.13 min per task including 5 dummy volumes to allow for T_1_-equilibration effects). For the 7 s ISI group (13 participants), we acquired 85 image volumes per task (4.36 min including 5 dummies). The TR (for both ISI groups) was chosen to maximise whole-brain coverage (including the whole of the cerebellum) and to ensure that slice-acquisition onset was offset with stimulus onset, allowing for distributed sampling of slice acquisition across the study (Veltman et al., 2003). Specifically, the four stimuli within each block were presented at acquisition slices 1, 28, 12, 39 when the ISI was 5 s, and at acquisition slices 1, 13, 25, and 37 when the ISI was 7 s. This ensures that different stages of the hemodynamic response are sampled within each brain region (i.e., the cerebellum in this study).

The acquisition of functional images for all 10 conditions took at least 33.88 min for participants with 5 s ISIs and at least 41.07 min for participants with 7 s ISIs (depending on time between sessions/runs). These times do not include out-of-scanner training, setting up and getting the participant into the scanner, collecting structural images, and unplanned technical hiccoughs. Participants took approximately 60–80 min to complete the study.

For anatomical reference, a high-resolution T_1_-weighted structural image was acquired after the participants completed the tasks using a 3D modified driven equilibrium Fourier transform (MDEFT) sequence (TR/TE/inversion time [TI] = 7.92/2.48/910 ms, flip angle = 16°, 176 slices, voxel size = 1 mm^3^).

### Analysis of Imaging Data

Data preprocessing and statistical analyses were performed using SPM12 (Wellcome Trust Centre for Neuroimaging, London, UK) in MATLAB 2012a. Standard SPM preprocessing was applied. After discarding the first five volumes (the dummy scans that allowed for equilibration effects), functional volumes were spatially realigned to the first EPI volume and unwarped to compensate for nonlinear distortions caused by head movement or magnetic field inhomogeneity.

We chose the unwarping procedure in preference to including the realignment parameters as linear regressors in the first-level analysis because unwarping already accounts for nonlinear movement effects by modelling the interaction between movement and any inhomogeneity in the T_2_* signal. After realignment and unwarping, we checked the realignment parameters to ensure that participants moved less than one voxel (3 mm) within each scanning run in any direction. They all did. The structural T_1_ image was co-registered to the mean EPI image, which had been generated during the realignment step and then spatially normalised to the Montreal Neurological Institute (MNI) space, using the unified normalisation–segmentation tool in SPM12. To spatially normalise all EPI scans to MNI space, we applied the deformation field parameters that were obtained during the normalisation of the structural T_1_ image. The original resolution of the different images was maintained during normalisation (voxel size 1 mm^3^ for structural T_1_ and 3 mm^3^ for EPI images). Following the normalisation procedure, functional images were spatially smoothed with a 6-mm full-width at half-maximum (FWHM) isotropic Gaussian kernel to compensate for residual anatomical variability and to permit application of Gaussian random-field theory for statistical inference (Friston et al., 1994).

#### First-level analysis

At the first-level statistical analyses, each preprocessed functional volume was individually inspected for oddities before being entered into a participant-specific and fixed-effect analysis, which used the general linear model (Friston et al., 1994). Each task was modelled as a separate run/session, with four regressors per task: One modelled instructions, while the others distinguishing between correct, incorrect, and missing responses. Onset times for all four regressors were modelled as single events, not blocks, to provide a more accurate model of the hemodynamic response (Mechelli et al., 2003). This was particularly important for the current paradigm given that the ISI was 5 s or 7 s and the response time was within 3 s. Stimulus functions were then convolved with a canonical hemodynamic response function. To exclude low-frequency confounds, the data were high-pass filtered using a set of discrete cosine basis functions with a cut-off period of 128 s, and the contrasts of interest were generated for each of the conditions of interest (relative to fixation).

#### Second-level analysis

A second-level ANOVA included the contrast images of correct responses from all 10 different conditions, each entered separately for each group of participants (one performing the paradigm with an ISI of 5 s, the other performing the paradigm with an ISI of 7 s). This resulted in 20 conditions in total. Modelling the two different cohorts (i.e., the 5 s and 7 s ISI groups) separately allowed us to test whether each effect of interest was consistent across ISI or differed according to whether the ISI was shorter (5 s) or longer (7s). This was tested with the interaction between each effect of interest and ISI (i.e., group), and no significant interactions were obtained.

The statistical contrasts used to identify each function of interest are described below and summarised in Table 3. For each function, there was a main (primary) contrast. In addition, to increase confidence that activation reflected the function of interest, we used the inclusive masking option in SPM. This limits the statistic output to voxels that are also activated in other contrasts. For example, to identify sentence effects that were common to different tasks, the main effect of sentences > nouns, across tasks, can be masked with the effect of sentences > nouns for each task. The significance threshold for the main contrast was set at a voxel level *p* threshold of <0.05, using family-wise error correction for multiple comparisons across the whole brain. The significance threshold for all inclusive masks was set at *p* < 0.001 uncorrected.

**Table 3.** Statistical contrasts used to identify each effect of interest and the functional localisers

Task number	1	2	3	4	5	6	7	8	9	10
Task type	Production				Rep		P-A Mat		Sem Assoc	
Stimulus type	S	N	Vb	C	S	N	S	N	V	A
Sentence processing										
Explicit sentence formulation	1	−1								
mask 1	1	−1			−1	1				
mask 2					−1	1	1	−1		
mask 3		−1	1							
Implicit sentence formulation	1	−1					1	−1		
mask 1	1	−1			−1	1				
mask 2					−1	1	1	−1		
Explicit sentence articulation	1	−1			1	−1				
mask 1	1	−1					−1	1		
mask 2					1	−1	−1	1		
Implicit sentence articulation	1	−1			1	−1	1	−1		
mask 1							1			
mask 2	1				1		−2			
Functional localisers for word processing										
1. Noun retrieval		4				−1		−1	−1	−1
mask 1		1				−1				
mask 2		1						−1		
mask 3		1							−1	
mask 4		1								−1
2. Event conceptualisation		−1	1							
3. Semantic association									1	1
mask 1									1	
mask 2										1
4. Auditory short-term memory									−1	1
5. Visual short-term memory									1	−1
6. Speaking / articulating words		1		1		1		−1	−1	−1

*Note*. Tasks numbers and type correspond to 1–10 in Figure 1. Production tasks are sentence production (S), noun production (N), verb production (Vb), and colour production (C). Rep = repetition; P-A mat = picture–auditory speech match, Sem Assoc = semantic association tasks. Stimulus type: S = sentences, N = nouns, Vb = verbs, C = colours, V = visual semantic association, A = auditory semantic association. 1 = the condition was given a positive weighting (activation) in the statistical contrast; −1 = the condition was given a negative weighting (baseline) in the statistical contrast.

##### Explicit sentence formulation.

The primary statistical contrast was sentence > noun production. The inclusive masks were interactions of sentence > noun for (i) production > repetition and (ii) production > matching (see Table 1). These interactions ensure that the effect of sentences (sentence > nouns) is significantly greater for production than repetition or matching.

##### Implicit sentence formulation.

The primary statistical contrast was sentences > nouns for production and matching (Tasks 1 and 7 > Tasks 2 and 8). The inclusive masks were interactions of sentence > nouns for (i) production > repetition and (ii) matching > repetition. These interactions ensure that the effect of sentences (sentence > nouns) is significantly greater for sentence production and matching than sentence repetition.

##### Explicit sentence articulation.

The primary contrast compared sentences to nouns for both production and repetition (Tasks 1 and 5 > Tasks 2 and 6). The inclusive masks were the interactions of sentence > noun for (i) production > matching and (ii) repetition > matching (see Table 1). These interactions ensure that the effect of sentences (sentence > nouns) is significantly greater for production and repetition than matching.

##### Implicit sentence articulation.

The primary statistical contrast was the main effect of sentences > nouns for production, repetition and matching (Tasks 1, 5, and 7 > Tasks 2, 6, and 8). The inclusive masks ensured that activation was higher for (i) auditory sentence-to-picture matching than fixation and (ii) sentence production and sentence repetition than auditory sentence-to-picture matching, as expected when activation reflects processing related to articulation (see Table 1).

#### Functional localisers

To probe the function of all the identified cerebellar regions in more depth, and understand the processing that is enhanced by sentences, we functionally localised the brain regions where activation increased with the demands on noun and verb retrieval, semantic associations, auditory short-term memory, visual short-term memory and speaking (single word articulation), independent of sentence formulation and articulation (i.e., excluding Tasks 1, 5, and 7 in Figure 1). We then looked to see whether activation associated with sentence formulation and/or articulation overlapped with activation associated with any of the functional localisers.

- **Noun retrieval** was identified where activation was higher for noun production (from pictures) compared to four corresponding noun tasks that did not require word retrieval (i.e., noun repetition, auditory noun-to-picture matching, visual semantic associations, and auditory semantic associations). The inclusive masks were the comparison of noun production to noun repetition (Task 2 > 6), auditory noun-to-picture matching (Task 2 > 8), visual semantic associations (Task 2 > 9), and auditory semantic associations (Task 2 > 10). See Table 1.
- **Event conceptualisation** was identified where activation was higher for verb production than noun production (Task 3 > 2).
- **Semantic association** was expected within areas where activation was higher for visual and auditory semantic associations (Tasks 9 and 10 in Figure 1) compared to fixation. The inclusive masks were each of the semantic association conditions compared to fixation independently. This ensured that the effects were related to processing that was independent of stimulus modality but did not exclude executive functions and finger pressing involved in making the response. See Table 1.
- **Auditory short-term memory** activation was expected within the set of regions that were more activated by auditory semantic association (hearing two nouns and deciding if they were semantically related or not) compared to visual semantic associations (Task 9 > 10), that involved the same task and object concepts without auditory input. There were no inclusive masks. See Table 1.
- **Visual short-term memory** activation was expected within the set of regions that were more activated by visual semantic association compared to auditory semantic associations, that involved the same task and object concepts, without pictorial stimuli (Task 10 > 9). There were no inclusive masks. See Table 1.
- **Speaking (articulating)** was identified where activation was higher for: noun production, noun repetition, and colour naming more than auditory noun-to-picture matching, visual semantic association, and auditory semantic association (Tasks 2, 3, and 6 > Tasks 8, 9, and 10). This activation includes articulatory planning that might occur implicitly during auditory sentence-to-picture matching and overt motor execution of speech which was not expected during any of the matching tasks.

## RESULTS

### In-Scanner Behaviour

All participants performed well and contributed a consistently high number of correct trials to all the effects of interest (mean accuracy was over 90% for all tasks). See Figure 2 for an overview of all accuracy and RT results.

RTs for the speech production tasks are interpreted with caution because of missing data from half the participants (see Materials and Methods). Nevertheless, the pattern of response in the available data corresponded to expectation with longer RTs for sentences than verbs (*t*_(33.46)_ = 2.16, *p* = 0.038) and for nouns than verbs (*t*_(29.23)_ = 2.20, *p* = 0.036), but no difference between sentence and noun production (*t*_(35.17)_ = 0.11, *p* = 0.91). These effects did not depend on ISI (*F*_(1.558, 21.815)_ = 0.394, *p* = 0.628, Huynh-Feldt corrected) because there was also no interaction effect between ISI (5 s vs. 7 s) and tasks (*F*_(1, 14)_ = 0.001, *p* = 0.97); see Figure 2.

### Neuroimaging Results

#### Regions involved in sentence formulation

Activation in bilateral cerebellum lobule VIIb (CbVIIb) and the left intraparietal sulcus was higher for sentence production than all other tasks, as shown in Figure 3 and Figure 4 (see also Table 4). This is consistent with processing related to sentence formulation. No cerebellar regions were significantly more activated by sentence > noun processing during auditory sentence-to-picture matching than auditory repetition. In other words, there was no evidence for implicit sentence formulation during the auditory sentence-to-picture matching task.

**Table 4.** Brain areas activated for sentences more than noun processing

Anatomical labels	Co-ordinates			Vx	Sent > Nouns/Verbs			Interactions			Speech	
	*x*	*y*	*z*	0.05 cor	P	R	M	Sent > Nouns			S*p* > M	aSM > Fx
								P > R	P > M	R > M		
**Sentence Formulation**	Z scores											
L Cb VIIb/Crus II	−24	−69	−45	4	6.1 / 4.9	–	–	4.5	3.6	–	5.6	2.5
R Cb VIIb	+27	−66	−45	12	5.6 / 6.2	–	–	3.8	3.1	–	7.4	3.6
L IPS	−36	−54	48	10	7.3 / –	–	–	3.9	3.2	–	–	4.9
**Sentence articulation**												
R Cb VIIIb	+15	−45	−51	8	5.8 / 4.6	5.0	–	–	4.1	3.6	5.0	–
**Sentences > Nouns, all tasks**												
R Cb Crus II	+18	−72	−42	13	6.5 / 5.3	2.9	3.0	–	–	–	6.0	3.8
L IFS	−51	+30	+21	21	5.0 / 4.7	3.5	2.7	–	–	–	–	3.8
L MFG	−48	+39	+12		5.1 / 3.8	3.0	1.8	–	–	–	–	3.3

*Note*. Sent = sentences, P = production tasks, R = repetition tasks, M = matching tasks, S*p* > M (speaking > matching) = noun production, noun repetition and colour naming > auditory noun-to-picture matching, visual semantic associations and auditory semantic association (i.e., Localiser 6); aSM = auditory sentence-to-picture matching task. Fx = fixation. IPS = intraparietal sulcus, IFS = inferior frontal sulcus, MFG = middle frontal gyrus. A dash indicates that the *Z* score was not significant at the specified threshold (see details of statistical contrasts in text).

#### Regions involved in sentence articulation

Activation in right cerebellum lobule VIIIb (CbVIIIb) was higher for sentence production and sentence repetition than all other tasks (Table 4). This area was not activated (*p* > 0.05 uncorrected) during any of the matching tasks including auditory sentence-to-picture matching (Figures 3 and 4). It was therefore consistent with sentence articulation. In addition, parts of the right posterior cerebellum (crus II), left inferior frontal sulcus and left middle frontal gyrus were more activated during all three sentence processing tasks than the corresponding noun conditions (Table 4), with greater activation during sentence production and sentence repetition than auditory sentence-to-picture matching (Figure 2).

#### Functional localisers

In all cerebellar regions associated with sentence processing, activation was also observed for speaking more than matching nouns in the absence of sentence processing (Localiser 6). There was no overlap between sentence processing regions in the cerebellum and regions associated with noun retrieval, event conceptualisation, semantic associations, or auditory or visual short-term memory (Localisers 1–5).

Figure 5 illustrates the cerebellar regions, outside the sentence processing regions, that were associated with speaking (Localiser 6) and semantic associations (Localiser 3). The areas associated with noun retrieval (Localiser 1) were within those associated with speaking (Localiser 6) but did not overlap with any of the regions associated with sentence processing. Event conceptualisation (Localiser 2) did not activate the cerebellum, but this is not because the verb production task was insensitive to event conceptualisation. As expected from previous studies of verb processing (Takashima et al., 2020; Willms et al., 2011), the left posterior middle temporal region [−54, −60, +3] was more activated for verb production than noun production (*Z* = 4.5, *p* < 0.001).

## DISCUSSION

Our functional neuroimaging analyses of sentence production have identified and dissociated three distinct cerebellar regions sensitive to the demands on sentence compared to noun processing: (1) Bilateral lobule VIIb was more activated by sentences than nouns during production, as expected for effects related to sentence formulation; (2) right lobule VIIIb was more activated by sentences than nouns during tasks involving overt speech (production and repetition) but not during auditory speech-to-picture matching, as expected for effects related to sentence articulation; and (3) right cerebellar Crus II was more activated by sentences than nouns for all three sentence tasks, though less for auditory sentence-to-picture matching than sentence production or sentence repetition.

As all sentence related regions were also activated during the production of nouns and verbs (i.e., during the speaking localiser), none of the identified regions could be described as specific to sentence production. Instead, their processing was involved in more generic speech production processing that was enhanced during sentence production. Below we discuss how our results fit with and extend prior findings, and their novel implications for understanding how the cerebellum contributes to both word and sentence production.

### Sentence Formulation

Our experimental plan defined sentence formulation as the processes involved in (i) finding a syntactic structure that assigns the roles of the two objects in the event to the roles of grammatical subject and grammatical object, and (ii) ordering the words to capture the intended meaning. We identified the brain regions associated with such processing by searching for regions that were more strongly activated for sentence production than sentence comprehension or sentence repetition, after controlling for noun processing in each task. Three regions were identified: the right lobule VIIb of the cerebellum (rCbVIIb), the left homologue extending into Crus II (CbVIIb-Crus II) and the left intraparietal sulcus.

Our observation of co-activation in cerebellar lobule VII and parietal cortices is consistent with some prior studies (Diedrichsen et al., 2019; Stoodley et al., 2012), particularly those investigating verbal working memory. For example, right lobule VIIb/VIIIa and left inferior parietal co-activated during the maintenance phase of verbal working memory (Chen & Desmond, 2005), right cerebellar lobule VIIb was found to functionally connect to the posterior parietal cortex during the late stages of verbal encoding (Macher et al., 2014), and right-lateralized cerebellar lobule VIIb/VIIIa has been shown to co-activate with left lateralised language regions during verbal working memory (Ng et al., 2016). While right-lateralized cerebellar lobule VIIb/VIIIa and left lateralised language regions have been associated with verbal working memory, bilateral cerebellar VIIb/VIIIa activation is associated with visual working memory (Ng et al., 2016). An explanation of CbVIIb activation in terms of verbal or visual short term memory does not, however, fit with our own findings.

In our study, the left and right cerebellar VIIb regions we associated with sentence formulation did not correspond to regions activated during either auditory or visual short-term memory (Localisers 3 and 4). Nor did they correspond to regions associated with verb production (Localiser 2), noun retrieval (localiser 1), or semantic associations (Localiser 3). More tellingly, the sentence formulation areas were located within the speech production network (Localiser 6) consistent with a meta-analysis of prior fMRI studies showing cerebellar lobule VIIb activation during speech production (Skipper & Lametti, 2021). We can rule out an interpretation in terms of overt motor execution of speech because (i) activation was higher for sentence production than sentence repetition, which also required exactly the same overt motor execution; and (ii) the cerebellar VIIb regions associated with sentence formulation (production > repetition or matching) were also activated, although to a much lesser extent, during auditory-sentence-to-picture matching, which does not require any overt speech production (see Table 4 and Figure 4).

A unifying explanation for this combination of findings would be that cerebellar VIIb activation, in our study, reflects the demands on sequencing (ordering) both words and phonemes during speech production. It is maximum for sentence production because both the words and phonemes need to be ordered. In contrast, during sentence repetition, the demands on phonetic or word sequencing are less because both the word and phonetic order are constrained by the auditory input. By word ordering, we are referring to how words need to be sequenced into a meaningful sentence with a constrained order—either *the noun is verbing the noun* (e.g., “The goat is eating the hat”) or *the noun is verbing preposition the noun* (“The zebra is drinking from the pool”). By phoneme ordering, we are referring to how phonemes need to be sequenced within a word (e.g., “cat” is /k/ /æ/ /t/ and not /t/ /æ/ /k/). Phoneme ordering determines the pronunciation so that the listener can perceive the intended word, while word ordering determines the syntactic structure and semantic meaning of a sentence. Both are fundamental aspects of language organization and comprehension.

Future studies are required to investigate (1) how the demands on word ordering during speech production influence bilateral cerebellar VIIb activation; (2) whether the co-occurring activation we observed in left intraparietal sulcus and cerebellar VIIb regions is task or condition dependent; and (3) whether there are multiple functionally distinct processing regions within lobule VIIb or (4) whether cerebellar VIIb activation associated with verbal and visual memory in prior studies overlaps with the cerebellar VIIb activation associated with sequencing words and phonemes in the current study.

### Sentence Articulation

Right cerebellar lobule VIIIb was the only region where activation was enhanced for sentences more than nouns in the production and repetition tasks but not in the matching tasks. This part of VIIIb was also enhanced for all speaking tasks with nouns but was not activated during silent auditory sentence-to-picture matching task compared to fixation (*p* > 0.05 uncorrected). Nor did it overlap with activation associated with the verb and noun retrieval, semantic, auditory memory, or visual memory (Localisers 1–5). This pattern of response is consistent with the demands on the overt execution of speech which will be higher when sentences rather than nouns are being articulated. We did not find any other fMRI papers that have associated VIIIb with speech production, perhaps because this region is often excluded from the field of view in fMRI scanning (*z*-MNI coordinate below −50 mm). Our results suggest that it should be included in fMRI studies of speech production and sentence processing.

### Sentence Processing in Right Cerebellum Crus II, Across Tasks

Here we discuss why activation in a paravermal part of right Crus II was more activated by sentences than nouns, across speaking and matching tasks. Recent meta-analyses (across studies) of cerebellar activation (Skipper & Lametti, 2021) as well as task comparisons within participants (King et al., 2019) have associated right cerebellar Crus II activation with listening to stories (Guell et al., 2018), speech perception (Skipper & Lametti, 2021) and semantic processing/word comprehension (King et al., 2019). In this context, the enhanced cerebellar activation that we observed for sentences more than nouns, across tasks, might be interpreted as reflecting enhanced semantic processing. However, neither of our own semantic localiser tasks (auditory and visual semantic association) activated the paravermal part of right Crus II where activation was enhanced by sentence processing across tasks. To the contrary, the functional localisers demonstrated that the paravermal right Crus II sentence processing region was within speech production regions (of single words, and not just sentences) and also activated by (silent) auditory sentence-to-picture matching, consistent with covert articulatory activity. The enhanced paravermal right Crus II activation that we observe for sentences than nouns across tasks may therefore reflect heightened demands on covert articulation that stem from the greater word count and syntactic complexity inherent in sentences compared to noun phrases.

We also considered the possibility that the paravermal right Crus II sentence processing region that we identified reflects the demands on working memory, as proposed by Küper et al. (2016) who found that paravermal rather than lateral parts of Crus II are sensitive to both visual and verbal working memory demands in tasks using letters and abstract shapes with no semantic content. Plausibly covert articulatory activity and/or the demands on working memory can explain right cerebellum Crus II activation during story listening (Guell et al., 2018), speech perception (Skipper & Lametti, 2021), and word comprehension (King et al., 2019). However, further studies are needed to determine whether different parts of Crus II (lateral and paravermal) are involved in covert articulation and/or working memory and/or sentence comprehension. The co-occurrence of activation in the right cerebellum Crus II with activation in the left inferior frontal sulcus and left middle frontal gyrus also requires further investigation. For example, (i) are all of these regions involved in covert articulation and/or working memory; and, (ii) does co-occurring activation depend on the task/conditions tested.

## SUMMARY AND CONCLUSIONS

In summary, our multi-task within-participant paradigm has allowed us to dissociate three distinct parts of the cerebellum where activation is enhanced during sentence production compared to noun production. As the same cerebellar regions were also activated during noun production, albeit to a lesser extent, they are not specifically involved in sentence processing. Instead, we argue that our paradigm has serendipitously distinguished three functionally distinct speech production regions in the cerebellum; each of which plays a particularly active role when the speech being produced involves sentences. As argued above, we propose that (1) bilateral cerebellar VIIb is involved in sequencing words and phonemes for speech production; (2) right cerebellar Crus II is involved in covert articulatory planning; and (3) right lobule VIIIb is involved in overt motor execution of speech. These findings not only provide more specific hypotheses for interpreting cerebellar activation during speech production, they also highlight and explain the enhanced demands of the cerebellum during sentence production.

## ACKNOWLEDGMENTS

We thank Eldad Druks for creating the picture stimuli.

## FUNDING INFORMATION

Cathy J. Price, Wellcome Trust (https://dx.doi.org/10.13039/100010269), Award ID: 097720/Z/11/Z. Oiwi Parker Jones, Medical Research Council (https://dx.doi.org/10.13039/501100000265), Award ID: MR/X00757X/1.

## AUTHOR CONTRIBUTIONS

**Oiwi Parker Jones**: Conceptualization: Equal; Formal analysis: Equal; Writing – original draft: Equal. **Sharon Geva**: Conceptualization: Supporting; Writing – original draft: Equal. **Susan Prejawa**: Conceptualization: Supporting; Data curation: Lead; Formal analysis: Supporting; Writing – review & editing: Supporting. **Thomas M. H. Hope**: Methodology: Supporting; Writing – review & editing: Supporting. **Marion Oberhuber**: Conceptualization: Supporting; Data curation: Supporting; Writing – review & editing: Supporting. **Mohamed L. Seghier**: Conceptualization: Supporting; Formal analysis: Supporting; Methodology: Supporting; Writing – original draft: Supporting. **David W. Green**: Conceptualization: Supporting; Supervision: Equal; Writing – original draft: Equal. **Cathy J. Price**: Conceptualization: Lead; Data curation: Supporting; Formal analysis: Equal; Funding acquisition: Lead; Investigation: Lead; Supervision: Equal; Writing – original draft: Equal.

## DATA AND CODE AVAILABILITY STATEMENT

Group level maps are available online at OSF (https://osf.io/6xgrm/) and include the main contrasts in the article (sentence formulation, sentence articulation, and implicit speech) plus the six localisers we used for interpretation.

## TECHNICAL TERMS

Events: Events in the world (e.g., a goat eating a hat) can be described by sentences (“The goat is eating the hat”).
Sentence formulation: Involves lexical retrieval, syntactic ordering, and morphological agreement, which we assume bridges event conceptualisation and sentence articulation.
Sentence articulation: Refers to the overt production of a sentence.
Event conceptualisation: Refers to the semantic understanding of an event, which is assumed to be similar for both speech comprehension and production.
Semantic association tasks: Tasks that require participants to decide if two objects are strongly associated (cowboy and horse) or not strongly associated (hat and horse).
Production tasks: Tasks involving overt speech production (e.g., sentence production, naming); here excludes auditory repetition tasks, which we treat separately.
Auditory repetition tasks: Tasks that involve hearing speech (e.g., sentences or phrases) and then overtly producing the heard speech.
